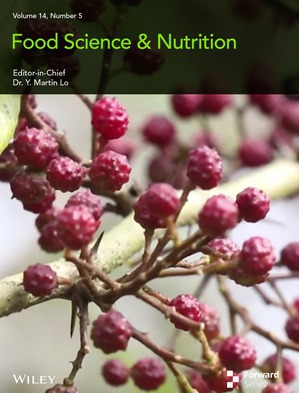# Cover Image

**DOI:** 10.1002/fsn3.71922

**Published:** 2026-05-26

**Authors:** Dipak Paudel, Santosh Koirala, Dhaka Ram Bhandari, Achyut Adhikari, Bhoj Raj Poudel, Megh Raj Pokhrel

## Abstract

The cover image is based on the article *Temporal Dynamics of Phytochemicals in Selected Medicinal Plants Across Gandaki Province, Nepal* by Dipak Paudel *et al.,* https://doi.org/10.1002/fsn3.71802.